# Perirenal Adipose Tissue—Current Knowledge and Future Opportunities

**DOI:** 10.3390/jcm10061291

**Published:** 2021-03-21

**Authors:** Adriana Grigoraș, Raluca Anca Balan, Irina-Draga Căruntu, Simona Eliza Giușcă, Ludmila Lozneanu, Roxana Elena Avadanei, Andreea Rusu, Laura Adriana Riscanu, Cornelia Amalinei

**Affiliations:** Department of Morphofunctional Sciences I, “Grigore T. Popa” University of Medicine and Pharmacy Iasi, Iasi 700115, Romania; raluca12usa@yahoo.com (R.A.B.); irinadragacaruntu@gmail.com (I.-D.C.); simonaelizagiusca@gmail.com (S.E.G.); ludmila_liliac@yahoo.com (L.L.); roxanavadanei@yahoo.com (R.E.A.); andreeadima27@yahoo.com (A.R.); laura_knieling@yahoo.com (L.A.R.)

**Keywords:** perirenal adipose tissue, chronic kidney disease, cardiovascular diseases, obesity

## Abstract

The perirenal adipose tissue (PRAT), a component of visceral adipose tissue, has been recently recognized as an important factor that contributes to the maintenance of the cardiovascular system and kidney homeostasis. PRAT is a complex microenvironment consisting of a mixture of white adipocytes and dormant and active brown adipocytes, associated with predipocytes, sympathetic nerve endings, vascular structures, and different types of inflammatory cells. In this review, we summarize the current knowledge about PRAT and discuss its role as a major contributing factor in the pathogenesis of hypertension, obesity, chronic renal diseases, and involvement in tumor progression. The new perspectives of PRAT as an endocrine organ and recent knowledge regarding the possible activation of dormant brown adipocytes are nowadays considered as new areas of research in obesity, in close correlation with renal and cardiovascular pathology. Supplementary PRAT complex intervention in tumor progression may reveal new pathways involved in carcinogenesis and, implicitly, may identify additional targets for tailored cancer therapy.

## 1. Introduction

Adipose tissue is a dynamic cellular complex that includes three distinct cell types: white, brown, and beige (“brite”), each of them displaying a particular morphofunctional profile. These cells are organized in humans into four different types of fat tissues: white, brown, beige, and perivascular adipose tissue [1]. All of these fat deposits contain adipocytes, vascular and nerve structures, preadipocytes, pericytes, and immune cells (mostly connective tissue mast cells) [1]. 

These fat deposits are physiologically involved in the maintenance of local and general homeostasis, via their endocrine and paracrine activity, but they may intervene in the pathogeny of some diseases. In this respect, the role of liver and muscle adipocytes in the development of diabetes mellitus [2], the intervention of epicardial fat in the development of atherosclerosis and ischemic coronary disease [3], and the involvement of perivascular adipose tissue (PVAT) in the pathogenesis of hypertension [4] have been recently identified.

Human fat is predominantly represented by white adipose tissue, which is organized in subcutaneous and visceral adipose tissue. Visceral white adipose tissue consists of gonadal fat deposits, epicardial adipose tissue, retroperitoneal, mesenteric, omental fat depots, and perirenal adipose tissue (PRAT) [5,6]. PRAT is included, according to some authors, in the so-called “ectopic fat”, along with PVAT, pericardial adipose tissue, renal sinus fat, and adipose tissue located in different organs or tissues (e.g., muscle and liver) [7,8].

Currently, PRAT is considered a special visceral adipose deposit in terms of its specific anatomical features, regarding vascularization and innervation, in the context of its location in the proximity of the kidney [9]. The most accurate imagistic methods for PRAT size quantification are ultrasound, magnetic resonance imaging, and computer tomography [10]. These methods have practical applications, considering that PRAT with an increased size is associated with a high cardiovascular and/or metabolic risk [7,11]. 

PRAT is also a metabolically active tissue, considering its ability to produce a panel of adipokines or cytokines which regulate renal activity via paracrine or endocrine mechanism, such as: adiponectin, leptin, resistin, tumor necrosis factor-α (TNF-α), visfatin, interleukin-6 (IL-6), and IL-1β [9]. Additionally, recent evidence of PRAT involvement in local tumor invasion has been reported [9].

In this context, our article reviews the most recent data regarding PRAT origin, structure, and its involvement in different pathological conditions, knowledge that may represent a keystone for new approaches in renal, metabolic, and cardiovascular diseases therapy and in prevention of local tumor progression.

## 2. Origin and Structure 

PRAT is located around the kidney and the adrenal gland, in the retroperitoneal space, between the renal capsule and the renal fascia (Gerota’s fascia) [9,12], while paranephric fat is adjacent to PRAT, the renal fascia being located between these two fat areas [9,12,13]. The renal sinus fat, a deposit of adipose tissue located at the medial border of the kidney, is associated with calyces, renal vessels, nerve fibers, and lymphatic channels of this compartment. Due to its relationship with the renal vessels, renal sinus fat is considered to act as PVAT, being mainly involved in blood pressure control [14,15]. Morphologically, the paranephric fat is composed predominantly of cells exhibiting unilocular-type lipid inclusions, while PRAT is mainly consisted of brown adipocytes [12,16]. 

The arteries that provide PRAT oxygen and nutrient intake derive from the branches of the left colic, lower adrenal, renal, lumbar, and ovarian or testicular arteries and generate an abundant anastomosing capillary network [17]. Lymphatic vessels that drain PRAT open in the renal subcapsular lymphatics and in para-aortic lymph nodes [18].

PRAT is not only well-vascularized, but is also richly innervated by branches of the ipsilateral celiac superior and inferior mesenteric ganglia, adrenal, gonadal, aorticorenal, and L1-L3 sympathetic trunk ganglia [19,20]. 

Although the studies on PRAT origin are limited, recent observations on PRAT adipogenesis have revealed that adipocyte precursors (preadipocytes) from the perirenal area are negative for endothelial markers, like CD31, or for hematopoietic stem cells, such as CD45, but express CD90 and CD166 positivity [16].

Morphologically, PRAT consists of a mixture of white and brown adipocytes, with most brown adipocytes in a dormant status [6,16,21]. These white and brown cells are associated with mesenchymal stem cells, preadipocytes, several inflammatory cells, along with many capillaries and nerve endings [9]. PRAT is considered a reservoir of mesenchymal stem cells (MSCs), which display the same phenotype as those obtained from other fat tissue depots and exhibit the capacity of differentiation into adipocyte, osteogenic, chondrogenic, and epithelial lineages [6], being a focus of research in regenerative medicine. These cells may show an immunoregulatory phenotype in response to inflammatory factors, such as IL-1β, IFNγ, TNF-α, which may be produced by different immune cells and this ability may be exploited in anti-inflammatory therapy [6]. Moreover, TNF-α stimulates the secretion of IL-6 and IL-8 by these MSCs, followed by angiogenesis stimulation in experimental models, while IL-6, IFNγ, and TNF-α increase their immunosuppressive abilities in vitro [6]. These recent findings may represent a potential immunomodulation mechanism which may be used to enhance the therapeutic effectiveness in different types of inflammatory diseases, tissue injuries [6], cardiovascular, and renal diseases. 

PRAT consists predominantly of brown adipocytes, while white adipocytes form only a thin cellular layer at its periphery, in fetuses and babies (1–11 months of life) [10]. Thus, a progressive conversion to the unilocular white adipocytes is carried out in PRAT, brown adipocytes being represented only by small cellular islands, dispersed within a white adipose area. Most PRAT is thought to be made up of dormant brown adipocytes, while active brown adipocytes are rare in adults, these being located in areas which contain a high number of sympathetic nerve endings [16].

An analysis of gene expression in various human fat depots revealed that PRAT is analogous to subcutaneous adipose tissue, being different from that of visceral adipose tissue [22]. The differences between PRAT and subcutaneous adipose tissue consist in expression of RNA binding motif single stranded interacting protein 1 (RBMS1), ankyrin repeat domain 20 family member A1 (ANKRD20A1), and DnaJ heat shock protein family (Hsp40) member B1 (DNAJB1) [21,22]. 

Recent studies have shown that approximately 30% of the PRAT population strongly expresses UCP-1, 20% of them having a single-locular phenotype, while the others exhibit a multilocular appearance [16,23]. This population does not express LIM homeobox 8 (LHX8), like brown adipocytes in other locations, but expresses a different panel of genes, such as mitochondrial creatine kinase 1 and 2 (CKMT1/2), carbonic anhydrase 12 (CA12), transglutaminase 2 (TGM2), arginase 2 (ARG2), 3-hydroxy-3-methylglutaryl-CoA synthase 2 (HMGCS2), cordon-bleu WH2 repeat protein (COBL), potassium two pore domain channel subfamily K member 3 (KCNK3), PPARG coactivator 1 alpha (PGC1-α), adrenergic receptor beta 3 (ADRβ3), Acyl-CoA thioesterase 11 (ACOT11), retinoid X receptor gamma (RXRγ), muscle associated glycogen phosphorylase (PYGM), fatty acid binding protein 3 (FABP3), homeobox C9 (HOXC9), and neurotrophic receptor tyrosine kinase 3 (NTRK3) [21,22,24,25]. There is also a phenotypic variability according to their location. Accordingly, UCP-1 positive multilocular perirenal adipocytes are located around the adrenal gland, in the hilum and convexity of the kidney, and much less often at the lower pole of the kidney, while UCP-1+ unilocular perirenal adipocytes are evenly distributed within PRAT [16].

The morphological variability is also gender-dependent, PRAT being much more developed in men compared to women, without a direct relationship between the body mass index (BMI) and its volume [26]. These findings were reported after computed tomography (CT) measurements were carried out on 123 persons (58 women, with an average age of 59 years and mean BMI of 28.9 kg/m^2^ and 65 men with an average age of 60.0 years and mean BMI of 28.9 kg/m^2^) [26]. The same results have been obtained by Favre et al., in a study carried out on 40 patients (16 women and 24 men), with an average age of 57.6 +/− 18.1 years and mean BMI of 28.9 +/− 2.9/kg/m^2^ [27]. Furthermore, they observed that men had a higher PRAT volume at a comparable waist circumference [26]. PRAT gender variability is also manifested by its thickness and volume correlation with the waist circumference, in men, and its negative correlation with the thickness of subcutaneous fat tissue, in women [27].

PRAT gender differences are equally reflected in its histologic pattern. Thus, brown-like adipocytes with an increased expression of UCP-1 mRNA represent 33% in female and only 7% in male PRAT [28]. Although the PRAT “browning” mechanism after cold exposure is partially explained, it has been already observed that the resulted heat is rapidly dispersed throughout the body, a finding easily attributed to the abundance of kidney blood flow, as the kidneys receive about 20% of the cardiac output [28]. This phenomenon may be partially attributable to the anatomical association with the adrenal gland, which induces an intense “browning” of PRAT, by production of catecholamines [23,29].

Additionally, the stronger female PRAT ability to induce “browning” seems to be more likely related to the specific characteristics of the sex-related MSCs of this area and less likely to the direct intervention of sex hormones [28]. These findings are based on the results of a study conducted on a murine model that showed that Y-chromosome suppresses brown adipose tissue (BAT) UCP-1 expression [30]. 

## 3. PRAT in Chronic Renal Pathology

Due to its anatomical location, PRAT’s size increase may lead to chronic kidney damage, with a direct correlation between the thickness of this adipose tissue deposit and the kidney damage [31]. According to this observation, the ultrasound evaluation of PRAT volume is nowadays proposed as a parameter for the assessment of early renal lesions associated with obesity [32]. This observation is also supported by the high occurrence of proteinuria (almost tripled in people with BMI > 25 kg/m^2^) [33,34].

The mechanism of PRAT involvement in chronic kidney damage is not completely elucidated but it has been postulated that PRAT’s increase may result in a direct obstruction of renal parenchyma and vessels, followed by an increase of sodium reabsorption and, as a consequence, a high blood pressure, with alterations of renal functions in obese patients [10]. The direct compression of PRAT on renal parenchyma results in intra-renal pressure increase, associated with reduced blood flow rates in vasa recta [35,36]. As a consequence, an increased Na+ absorption in Henle’s loop, associated with a decreased NaCl delivery to the macula densa, results in low resistance in afferent arterioles, along with an increased glomerular filtration rate and activation of renin production by juxtaglomerular cells [35,36].

**Figure 1:**
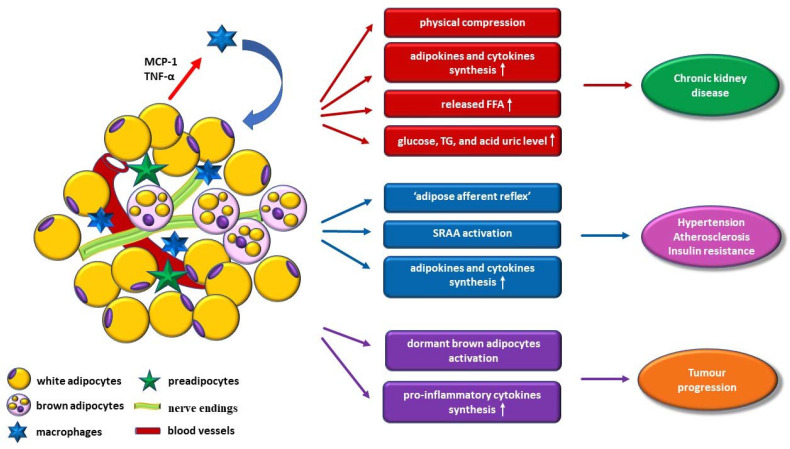
Schematic diagram of perirenal adipose tissue involvement in pathology. FFAs—free fatty acids; MCP-1—monocyte chemoattractant protein-1; SRAA—renin-angiotensin-aldosterone system; TGs—triglycerides; TNF-α—tumor necrosis factor-α; ↑—increased level.

In addition, PRAT compression of the renal parenchyma causes an increased interstitial hydrostatic pressure and a reduced renal blood flow, which result in stimulation of renin secretion, glomerular filtration, and tubular sodium reabsorption, respectively, all these processes accelerating the kidney disease progression (Figure 1) [10,31,36].

In this context, a recent study on 296 patients with hypertensive disease has shown that glomerular filtration rate reduction of <60 mL/minutes per 1.73 m^2^ is correlated to PRAT increase not to that of visceral adiposity, regardless of gender [37]. Moreover, a direct link between PRAT size and patients’ high serum uric acid and triglycerides, in chronic kidney disease [31] or with creatinin values, in hypertensive disease has been registered [37] as a consequence of glomerular filtration rate reduction [10].

The increased volume of visceral fat and, more specifically, of PRAT is associated with overproduction of free fatty acids, showing a serum level directly correlated with albuminuria [32,38]. Fatty acids metabolites, such as ceramides, have a direct renal lipotoxic effect [32,38]. Moreover, PRAT fatty acids excessive release induces an endothelial dysfunction, which is manifested by enhanced oxidation of tetrahydrobiopterin, followed by increased production of superoxides and decreased NO synthesis [10].

The cellular microenvironment of PRAT, characterized by the association between white and brown adipocytes, predipocytes, and macrophages, together with numerous nerve endings and blood vessels, is involved in insulin resistance, chronic kidney disease, hypertension, atherosclerosis, and, recently, in tumor progression. 

Excessive PRAT releases pro-inflammatory adipokines and chemokines, such as leptin, adiponectin, vaspin, resistin, interleukin-6 (IL-6), IL-1β, and TNF-α, which may influence the renal activity in a paracrine manner [9,39]. Thus, studies conducted on murine models showed that TNF-α and leptin synthesized by perirenal adipocytes are triggers for the development of renal fibrosis [27,40,41]. Concomitantly, leptin causes the remodeling and proliferation of the glomerular capillary endothelium, by activating the p38 mitogen-activated protein kinase (MAPK) pathway, while TNF-α induces endothelial dysfunction of the renal vessels, thus interfering with the glomerular filtration [42,43]. Leptin secretion also increases the sympathetic renal nervous activity, by stimulating the central nervous system proopiomelanocortin–melanocortin 4 receptor pathway [36].

Additionally, the reduction of the inflammatory profile of perirenal adipocytes, expressed by decreased levels of inflammatory cytokines including IL-1β, IL-6, and TNF-α, due to stimulation of heme oxygenase system associated with a decreased macrophage infiltration, results in an improved renal activity [10,44]. 

An important recent study has demonstrated a direct correlation between age and inflammatory phenotype of donor-derived stromal vascular fraction of perirenal adipose tissue (PRAT-SVF), expressed by a local recruitment of natural killer (NK) cells, which display a CD45+CD3-CD56+ phenotype. The proportion of NK cells in PRAT-SVF is associated with NKG2D receptor activation and transcripts encoding INFγ, suggesting that NK cells may be actively involved in pro-inflammatory mechanisms leading to functional impairment in elderly transplanted patients [45].

Current data have shown that PRAT size potentiates the lesions produced by other renal metabolic factors, such as abnormal insulin serum levels and increased glucose resistance or high triglycerides and uric acid levels, all these features being observed in patients with chronic kidney disease [10,46]. Furthermore, according to the results of a recent study, an increase of PRAT in patients with calcium phosphate apatite or uric acidic nephrolithiasis has been noticed [47]. Since it could not be specified whether there is a direct relationship between the occurrence of these lesions and PRAT volume, further research is needed to clarify this finding [47].

## 4. PRAT in Metabolic and Cardiovascular Pathology

Obesity is a pathological condition associated with cardiovascular risk, type II diabetes mellitus, dyslipidaemia, and high blood pressure. The deposition of triglycerides in ectopic adipose tissue, including PRAT, is attributed to an “exceedance” of the subcutaneous white adipose tissue storage capacity [48].

An increased waist circumference size is considered by some guidelines as a factor associated with the cardiovascular risk [49]. Moreover, its value is also providing general information about the size of the subcutaneous and perivisceral adipose areas [7,10]. 

During the last years, research has shown that the cardiovascular risk is more closely correlated with visceral fat tissue volume, including PRAT, in comparison to subcutaneous fat size [7,31,50]. In this regard, a recent study, conducted on a group of 702 overweight prepubertal children, revealed a relationship between PRAT size and carotid intima-media thickness [51]. Furthermore, the ultrasound evaluation of the thickness of the perirenal and epicardial fat areas may be considered useful in cardiovascular risk assessment, considering the strong relationship between the epicardial and PRAT volume and the carotid intima-media thickness detected in a group of healthy prepubertal children [39].

These data are supplemented by the study of Ricci et al. which reported a larger PRAT volume evaluated by ultrasound in morbidly obese male patients with BMI ≥ 40 or ≥35 kg/m^2^ (mean values of 15.6 ± 4.9 mm), compared with female counterparts (11.6 ± 4 mm) [11]. 

Despite these findings, the intimate mechanisms of PRAT involvement in cardiovascular etiopathogeny remain incompletely explained. According to literature, PRAT directly regulates the activity of the cardiovascular system by an “adipose afferent reflex”, which increases the blood pressure, as a result of enhanced renal sympathomimetic outflow induced by amplified afferent signals from fat deposits [52]. However, PRAT whitening in adult animals has been tested by administration of 6-hydroxydopamine (6-OHDA), a dopamine-derived sympathetic neurotoxin, and resulted in decreased sympathetic innervation and inhibition of adipose tissue browning, showing that PRAT development is a sympathetic-independent process [9]. 

Additionally, PRAT excess induces the activation of the renin-angiotensin-aldosterone system due to the compression of blood and lymphatic vessels, along with ureters, which may be responsible for the development of hypertensive disease, atherosclerosis, and insulin resistance (Figure 1) [7,53]. There is evidence that glomerular activity, which is important in homeostasis and normal blood pressure control, is influenced by PRAT size [7,53]. The association between hypertensive disease and PRAT size, regardless of other fat deposits indices, is supported by other studies, with direct correlation between excessive PRAT volume and the reduction of glomerular filtration rate [10,37]. Moreover, according to the study of Ricci et al., patients with hypertensive diseases have a larger PRAT thickness (average value of 13.6 mm), in comparison to normotensive patients (average value of 11.6 mm) [11], with additional correlation to age, anthropometric data (waist circumference and BMI), systolic blood pressure, insulin resistance, and glycated hemoglobin values [11]. These findings are supported also by the study performed on 102 uncomplicated overweight and obese patients, which demonstrated a close relationship between PRAT size and systolic and diastolic blood pressure, along with serum triglycerides values [46]. Moreover, UCP-1 protein low expression has been detected in PRAT of obese patients with hypertensive disease, compared to normal subjects [21,54]. 

Recent analyses performed on patients with high-grade obesity have also shown a correlation between serum creatinine levels and PRAT thickness, in a context of so-called “obesity-related-glomerulopathy” [55,56]. From a morphologic point of view, this condition is characterized by glomerular hypertrophy, with or without focal and segmental glomerulosclerosis and proliferation of glomerular mesangial cells, which are induced by a disruption of PRAT hormone and cytokine secretions [11,57,58]. Furthermore, adipokines and cytokines synthesized by dysfunctional perirenal adipocytes have paracrine or autocrine effects on the cardiovascular system [9,10]. Despite all these findings, there are relatively limited data regarding the correlation between PRAT volume regression and possible hypertensive disease remission [7]. However, hypertension remission after bariatric surgery attributed to PRAT’s size decrease has been reported in patients with morbid obesity [11]. Thus, sleeve-gastrectomy performed on a group of 89 patients with morbid obesity and hypertensive disease led to a reduction of doses of antihypertensive drugs prescribed or a withdraw of drug administration in 16 patients that showed normal blood pressure after the surgery, and no need to start the administration of a drug-based therapeutic regimen in 48 patients [11].

Consequently, the ultrasound assessment of PRAT volume should be included in the evaluation of obese patients, in order to establish the risk of cardiovascular and chronic renal disease, using microalbuminuria as a useful indicator of microvascular lesions and early renal dysfunction [10,46]. 

Furthermore, excessive PRAT is associated with an increased insulin resistance and dyslipidaemia, which in turn lead to an increased cardiovascular risk and to an accelerated age-related decline of renal function [11,37,46,50]. Recent published data support the finding that plasminogen inhibitor-1 activator (PAI-1), a mediator of extracellular matrix (ECM) accumulation in diabetic nephropathy produced by perirenal adipocytes, is involved in the development of diabetic nephropathy and insulin resistance, by increasing the recruitment of immune cells in obese people [59]. It is well recognized that hypoxia due to PRAT enlargement induces lipolysis and acts as a local pro-inflammatory trigger [60,61]. As a consequence, a high increase of immune cells component, mainly composed of macrophages, along with mast cells, neutrophils, and lymphocytes occurs in fat tissue areas [61]. All of these processes are associated with an increase of local synthesis of pro-inflammatory cytokines, such as leptin, chemerin, resistin, visfatin, retinol binding protein 4 (RBP4), and lipocalin 2 (LCN2) [62]. Their pro-inflammatory action is counterbalanced by adiponectin and omentin, both exhibiting an anti-atherogenic and anti-inflammatory capacity [61]. Although the activity of PRAT inflammatory cells is poorly understood, an analysis of these cells in pigs with obesity-related metabolic dysfunction showed an increase of local infiltration with macrophages, associated with an increased TNF-α expression [43]. Another study performed on a murine model revealed that leptin direct injection into PRAT results in adipose afferent reflex activation, supporting its well-known capacity to induce renal vascular and endothelial damage [42]. 

Although important steps have been made in deciphering PRAT involvement in the development of cardiovascular and metabolic diseases, further investigations are necessary for the development of a new generation of therapeutic tools, based on adipocyte targets. In this regard, it has been recently observed that a fish oil-rich diet [63], short time-high frequency physical exercises [64], reduction of meal frequency to 1–2/day or exposure to low temperatures [65] induce a decrease of PRAT volume [66]. 

In an attempt to analyze if PRAT browning may combat obesity, it was revealed that brown adipocytes require a dense vascular network to provide their high energy consumption [67]. In cases of vascular insufficiency, mitochondrial dysfunction occurs, leading to systemic insulin resistance [67], suggesting that promotion of browning may open promising perspectives in the therapy of renal pathology, hypertension, inflammation, or in the control of the general metabolic status.

Moreover, the main research objectives in cardiovascular and metabolic diseases could be the identification of molecular factors actively involved in stimulation of PRAT dormant brown adipocytes in order to develop appropriate therapeutic and prevention approaches.

## 5. PRAT in Tumor Pathology 

Several large-scale studies have confirmed a significant association between obesity and cancer [68,69]. The dysfunctional adipose tissue is one of the sources of growth factors, cytokines, adipokines or extracellular matrix scaffolding, which support tumor cell growth in obese patients [69,70,71]. 

The current knowledge regarding the relationship between PRAT and tumor pathology is limited: few studies report the possible involvement of this adipocyte area in supporting local or general invasion of tumor cells. Considering the anatomical relationship between PRAT and kidney, a correlation between PRAT size and clear cell renal carcinoma (conventional) local progression and life expectancy has been demonstrated in a study conducted on a group of 174 patients [72]. Consequently, the imaging evaluation of PRAT volume may be useful in assessing the prognosis in clear cell renal carcinoma [72,73]. 

**Figure 2:**
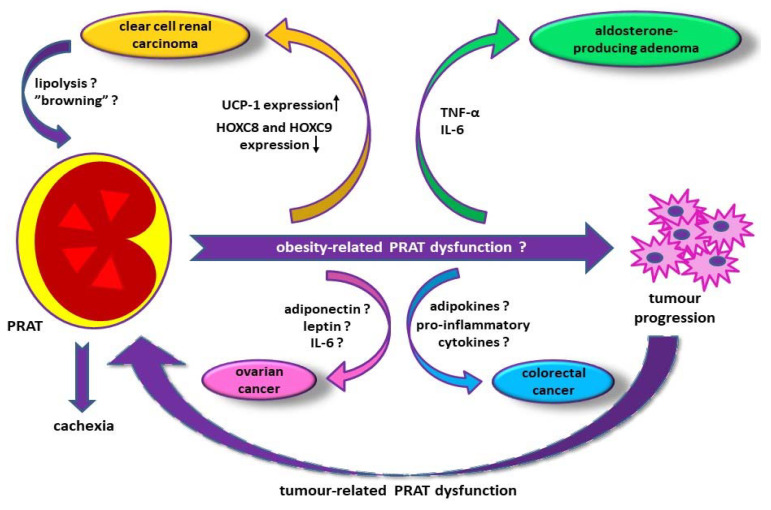
Hypothetical pathways of perirenal adipose tissue (PRAT) induced tumor progression. HOXC8—homeobox protein 8; HOXC9—homeobox protein 9; IL-6—interleukin 6; PRAT—perirenal adipose tissue; TNF-α—tumor necrosis factor-α; UCP-1—uncoupling protein 1; ↑—increased level; ↓—decreased level; ?—unknown.

Considering PRAT specific morphological profile, consisting of a mixture of white and brown adipocytes, it expresses high levels of UCP-1. In this context, an increased UCP-1 expression of PRAT is considered a negative prognostic factor in patients with clear cell renal carcinoma (Figure 2) [74]. Moreover, a decreased HOXC8 and HOXC9 genes expression, as a classical white adipocytes signature, has been detected in perirenal fat in patients with clear cell renal carcinoma when compared to healthy people, while TBX1, TMEM26, or CD137 expression was unchanged [74]. Furthermore, the result of the same study showed similar expression of white adipose cell markers, such as ADIPQ and LEP, in both study groups [74]. According to this data, a mechanism of PRAT “browning” occurs in patients with clear cell renal carcinoma [74]. Nonetheless, further investigations are necessary to complete the knowledge about the mechanism involved in PRAT regulation of metabolism and stimulation of tumor development [74].

The spectrum of factors involved in PRAT induced local tumor progression comprises overexpression of UCP-1 associated with perirenal adipocytes, underexpression of HOXC8 and HOXC9, and a possible added PRAT “browning” in clear cell renal carcinoma, promotion of tumor progression by adipokines and pro-inflammatory cytokines released by dysfunctional perirenal fat, cachexia due to brown adipocytes activation, and dedifferentiation of mature adipocytes at the invasive tumor front.

There are relatively limited data regarding the involvement of perirenal adipocytes in promoting ovarian tumor cells adhesion, migration, and invasion [75,76]. In this regard, PRAT stromal cells have been detected as stimulators of tumor growth [75,76]. According to the results of a recently published study on a group of 258 patients with stage III and IV ovarian cancer, PRAT thickness of more than 5 mm has been associated with a lower survival rate [77]. This finding supplements the observation that overexpression of adiponectin and leptin in patients with visceral obesity induces the progression of ovarian cancer and its recurrence, as a result of leptin-potentiated IL-6 synthesis and its contribution to survival of dormant tumor cells [78,79]. Furthermore, the immunosuppressive cytokines produced by PRAT, such as IL-10, along with the suppression of IL-6, IL-12p40, and CD86 result in stimulation of ovarian cancer progression, while stimulation of IFNγ, by abrogating IL-12 inhibition, leads to a favorable prognosis in malignant ascites [77].

Another mechanism contributes to a poor prognosis in ovarian cancer is that of increased UCP-1 activity in brown adipose tissue of PRAT, resulting in increased resting energy expenditure which results in tumor cachexia [77]. 

Beside ovarian and renal carcinoma, PRAT dysfunction has been also correlated to an increased risk and poor prognosis in colorectal cancer [80]. Furthermore, adipocytes located at the tumor invasive front gain a fibroblast-like phenotype, suggesting a preadypocyte population arising from dedifferentiated mature adipocytes, as a possible feedback loop resulting in PRAT dysfunction in abdominally metastasizing cancers [75]. 

PRAT is not only involved in tumor progression but, in addition, its large thickness is now considered as a predictor of post-surgery complications [81]. 

Furthermore, PRAT is an active metabolic tissue that releases a panel of inflammatory cytokines, such as TNF-α and IL-6, as a result of the increased number of macrophages in the excessive perirenal fat area [27]. In the same context, PRAT inflammatory profile associated with a local fibrosis induced by the overexpression of genes encoding fibronectin and type I collagen have been noticed in patients with aldosterone-producing adenoma, due to enhanced aldosterone production [54]. 

Although important steps have been made in deciphering the specific PRAT roles in tumor progression, the intimate molecular mechanisms of its involvement in carcinogenesis and tumor invasion are far from elucidation.

## 6. Conclusions

The past two decades have been marked by changes in the traditional perception of the adipose tissue structure and pathophysiology. Currently, PRAT is a particular visceral adipose deposit, with anatomical and morphological specific features related to its proximity to the kidney. The data concerning PRAT adipocytes origin and activity are limited, but recent research has been oriented towards several directions in order to exploit its potential in therapy and prevention of different diseases. 

Although PRAT displays a small size in comparison to that of subcutaneous or visceral fat deposits, the paracrine or autocrine mechanisms of action of the adipokines and pro-inflammatory cytokines which it produces maximize PRAT’s effects in the maintenance of renal and general homeostasis.

In this context, the main research objectives could be the identification of the regulation pathways of the molecular mechanisms involved in brown adipocyte lineage differentiation in PRAT, as the most promising therapeutic approach in cardiovascular diseases, chronic renal pathology, and tumor local progression.

## Data Availability

Not applicable.
